# Differential Coexpression Analysis Reveals Extensive Rewiring of *Arabidopsis* Gene Coexpression in Response to *Pseudomonas syringae* Infection

**DOI:** 10.1038/srep35064

**Published:** 2016-10-10

**Authors:** Zhenhong Jiang, Xiaobao Dong, Zhi-Gang Li, Fei He, Ziding Zhang

**Affiliations:** 1 State Key Laboratory of Agrobiotechnology, College of Biological Sciences, China Agricultural University, Beijing 100193, China; 2 Biology Department, Brookhaven National Laboratory, Upton, NY 11973, USA

## Abstract

Plant defense responses to pathogens involve massive transcriptional reprogramming. Recently, differential coexpression analysis has been developed to study the rewiring of gene networks through microarray data, which is becoming an important complement to traditional differential expression analysis. Using time-series microarray data of *Arabidopsis thaliana* infected with *Pseudomonas syringae*, we analyzed *Arabidopsis* defense responses to *P. syringae* through differential coexpression analysis. Overall, we found that differential coexpression was a common phenomenon of plant immunity. Genes that were frequently involved in differential coexpression tend to be related to plant immune responses. Importantly, many of those genes have similar average expression levels between normal plant growth and pathogen infection but have different coexpression partners. By integrating the *Arabidopsis* regulatory network into our analysis, we identified several transcription factors that may be regulators of differential coexpression during plant immune responses. We also observed extensive differential coexpression between genes within the same metabolic pathways. Several metabolic pathways, such as photosynthesis light reactions, exhibited significant changes in expression correlation between normal growth and pathogen infection. Taken together, differential coexpression analysis provides a new strategy for analyzing transcriptional data related to plant defense responses and new insights into the understanding of plant-pathogen interactions.

Plants as sessile organisms are subject to numerous attacks from microbes during their lifetime. As a result, plants have evolved a sophisticated immune system that enables each cell to monitor every invasion by microbes and to mount an appropriate defense response when necessary. Typical immune responses include the generation of reactive oxygen species, activation of the MAPK pathway, deposition of callose and the production of phytohormones, involving complicated transcriptional reprogramming^1^,^2^,^3^. These immune responses are interconnected and collaborative for resisting pathogens.

Microarray technology has provided a powerful approach for analyzing genome-wide gene expression profiling during plant immune responses. Typically, differentially expressed genes (DEGs) during plant immune responses are identified as potential plant defense-related gene candidates. Differential expression analysis considers each gene individually, while their potential interactions are ignored. However, genes or their protein products do not act in isolation; instead, they are interrelated with each other and act in close coordination^4^,^5^,^6^. Taking interactions between genes into account, various approaches based on gene sets^7^, coexpression^8^,^9^,^10^, machine learning^11^ and biological networks^12^ have been adopted to analyze transcriptional datasets involved in plant immunity^13^,^14^,^15^. For example, a large-scale immune coexpression network was constructed to identify immune-related functional modules involved in plant defense^10^. Previously, we employed an advanced machine learning method to integrate *Arabidopsis* gene networks with transcriptome data^11^. Through comprehensive network analysis, we revealed shared and distinct network organizations between pattern-triggered immunity and effector-triggered immunity in *Arabidopsis*.

Recently, differential coexpression analysis is emerging as an important complement to the traditional differential expression analysis^16^,^17^,^18^. Instead of focusing on individual genes, the goal of differential coexpression analysis is to identify gene pairs with different expression correlation between two conditions (such as healthy and diseased samples)^16^. One of the possible mechanisms behind differential coexpression is that the regulation of a pair of genes may be disrupted/activated under a certain condition^17^,^19^. A number of approaches have been developed to detect differential coexpression. Some methods focus on the detection of differential coexpression gene sets^20^,^21^, whereas others aim to identify differential coexpression gene pairs^22^,^23^. In 2013, Amar *et al*. developed a method called DICER to detect differentially coexpressed gene sets using a probabilistic model^20^. DICER not only identifies a gene set with correlation significantly altered between two conditions but also provides pairs of gene sets with changed correlational relationships. Based on Fisher’s z-test, Fukushima *et al*. explored tomato gene function via differential coexpression analysis^24^. Later, they published an R package called DiffCorr to identify differential coexpression gene pairs^22^. Yang *et al*. implemented several methods in the R package DCGL to identify differential coexpression gene pairs, differentially coexpressed genes (DCGs) and transcription factors (TFs) that regulate differential coexpression^23^.

Differential coexpression analysis has been widely used to analyze human disease-related transcriptional data^16^,^17^. Choi *et al*. applied differential coexpression analysis to detect major cellular changes in tumor cells and found gene groups whose co-regulation may contribute to malignant transformation^25^. Hudson *et al*. successfully inferred myostatin as the gene containing the causal mutation of their interested phenotype through differential coexpression analysis^26^. Recently, Reznik and Sander studied differential coexpression across metabolic pathways in both breast and kidney tumors, and they integrated regulatory information to study the drivers (*i.e.*, TF) of these changes^27^.

Although a plethora of transcriptional datasets measuring *Arabidopsis* gene expression during plant immunity are available^28^, to the best of our knowledge no study has been conducted to analyze plant immune responses using differential coexpression analysis. *Pseudomonas syringae* is a Gram-negative bacterial pathogen that causes diseases in a wide range of plant species. The *Arabidopsis*-*P. syringae* interaction is recognized as one of the most important model systems for understanding plant-pathogen interactions^29^. Transcriptomics studies focusing on this model system have already broadened our understanding of plant-pathogen interactions. For example, de Torres-Zabala *et al*. generated a high-resolution time series expression profile [Gene Expression Omnibus (GEO) accession number: GSE56094] measuring *Arabidopsis* gene expression following either mock, *P. syringae* pv. *tomato* DC3000 or *P. syringae* pv. *tomato* DC3000 *hrpA* challenges. They analyzed the expression changes of nuclear-encoded chloroplast-targeted *Arabidopsis* genes and showed that chloroplast was a key component of early immune responses^30^. In combination with hormone profiling, reverse genetics and RNA-seq analyses, they also explored the dynamics, interaction and contribution of jasmonic acid, coronatine (COR) and jasmonate ZIM-domain (JAZ) proteins to *P. syringae* disease progression^31^. Moreover, Lewis *et al*. conducted a comparative analysis on the same transcriptional data for exploring the transcriptional dynamics during microbial-associated molecular pattern-triggered immunity induced by *P. syringae* pv. *tomato* DC3000 *hrpA* treatment and effector-triggered susceptibility caused by *P. syringae* pv. *tomato* DC3000 challenge^32^.

To date, the GEO database^33^ contains over 400 samples related to *Arabidopsis* immune responses to the infection of *P. syringae*, which provides a great opportunity to revisit the transcriptomics data through some new computational methods and thus convert these data into new biological discoveries. In this work, we focused on two sub-series from GSE56094. We first showed that differential coexpression was a common phenomenon during plant immune responses. Then, we identified 1,315 genes (*i.e.*, DCGs) that frequently changed their coexpression partners. To explore the biological significance of differential coexpression, we identified some potential TFs regulating differential coexpression in plant immune responses with the assistance of known *Arabidopsis* gene regulatory networks. Furthermore, we investigated differential coexpression in the context of metabolic pathways. These results further indicated that the *Arabidopsis* gene network has been extensively rewired in response to infections by plant pathogens.

## Results and Discussion

### Differential coexpression is extensive during plant immune responses

**Figure 1:**
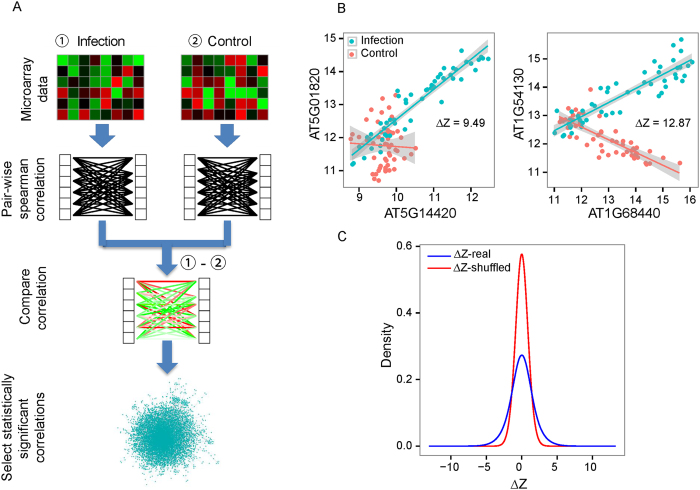
Overview of the differential coexpression analysis. **(A)** The computational framework used to identify differential coexpression gene pairs. **(B)** Two real cases of differentially coexpressed gene pairs. The *x*-axis and *y*-axis reflect gene expression level of the differentially coexpressed gene pairs. The solid lines reflect the linear correlation between two genes. **(C)** The distribution of transformed difference in correlation (Δ*Z*) in real (blue) and shuffled (red) data. Statistical analysis shows that the absolute values of Δ*Z* in real data are significantly larger than values in shuffled data (Student’s *t*-test, *p*-value < 2.2 × 10^−16^).

The microarray data GSE56094 is composed of 156 distinct samples from 13 time points in three conditions: mock treatments or infections by either virulent *P. syringae* pv *tomato* DC3000 or the corresponding nonpathogenic *hrpA* mutant, with four replicates for each condition^30^,^31^,^32^. In our work, we focused on mock and virulent *P. syringae* pv *tomato* DC3000 treatments. Therefore, 104 samples from GSE56094 were used in this work, including 52 samples from mock-treated control and 52 samples infected by bacteria (Fig. 1A). We only used 6,775 genes with an expression variance larger than 0.2 in either of the two conditions. The R package DiffCorr^22^ was used to identify differentially coexpresssed gene pairs. Briefly, Spearman correlation coefficients (SCCs) and associated *p*-values for all possible gene pairs among these 6,775 genes were calculated for each condition. Then, the difference in correlation between two conditions for every gene pair was further evaluated using Δ*Z*. A pair of genes is defined as differentially coexpressed if the expression correlations of the gene pair under two conditions (pathogen infection and mock treatment) are significantly different (see the Materials and Methods section for details). In total, we obtained 124,115 differential coexpression gene pairs among 4,748 genes (The complete list of these differential coexpression gene pairs is available at: http://systbio.cau.edu.cn/SI/index.php). Figure 1B illustrated two examples of differentially coexpressed gene pairs from our work. In the first example, the strength of coexpression between AT5G14420 and AT5G01820 increased from −0.03 to 0.96 after infection. With respect to the AT1G68440 and AT1G54130 gene pair in the second example, the strength of coexpression was reversed between control (−0.83) and infection (0.89).

To investigate whether these drastically changed coexpression patterns are biologically significant, we followed the strategy of Amar *et al*.^20^ to shuffle data and then assessed the distribution of coexpression differences for all possible gene pairs on the microarray. The coexpression difference for a gene pair is measured using Δ*Z* (Eq. 1, see the Materials and Methods section for details). A larger absolute value of Δ*Z* indicates a larger coexpression difference. If the differential coexpression is common, the real biological data will have larger coexpression differences than the shuffled data. We permuted the 104 samples by shuffling sample labels (*i.e.*, infection or control) and calculated Δ*Z* based on the shuffled data. As expected, the absolute values of Δ*Z* are larger in real data compared with shuffled data (Student’s *t*-test, *p*-value < 2.2 × 10^−16^, Fig. 1C). To test whether this phenomenon was prevalent during plant immune responses or it is specific to the microarray dataset we used, we repeated the analysis on a combined gene expression dataset generated by several different studies (GEO accession number: GSE5685, GSE5615, GSE21920, GSE18978 and GSE17500^34^) and found that the absolute values of Δ*Z* are larger in real data compared with shuffled data (Student’s *t*-test, *p*-value < 2.2 × 10^−16^, Figure S1A and Table S1). Furthermore, the same pattern was observed not only for gene expression under the infection of *P. syringae* but also for other pathogens, such as *Golovinomyces orontii* (GEO accession number: GSE5686, Student’s *t*-test, *p*-value < 2.2 × 10^−16^, Figure S1B, Table S1) and *Botrytis cinerea* (GEO accession number: GSE29642^35^, Student’s *t*-test, *p*-value < 2.2 × 10^−16^, Figure S1C). Based on the above analyses, we concluded that differential coexpression was a common phenomenon during plant immune responses.

### DCGs are heavily involved in plant immune responses

In our analysis, we defined genes that were frequently differentially coexpressed with other genes as DCGs. To identify DCGs, we tested the enrichment of differentially coexpressed gene pairs for each gene. For the gene *i* that participated in *k*_*i*_ differential coexpression gene pairs, the *p*-value was calculated based on a binomial probability model (see Eq. 2 in the Materials and Methods section). If the corrected *p*-value of gene *i* was smaller than a given cutoff of 0.05, gene *i* was assigned as a DCG. According to the criterion, we totally obtained 1,315 DCGs (Table S2).

Gene Ontology (GO)^36^ enrichment analysis of 1,315 DCGs revealed that the most significantly enriched GO term was “response to stimulus” (hypergeometric test, *p*-value = 9.80 × 10^−20^, Table S3A), indicating the extensive involvement of DCGs in plant responses to stimuli. Other biological processes related to plant defense responses have also been enriched in DCGs, such as “defense response” and “cell wall modification”. In addition to GO enrichment analysis, we compared 1,315 DCGs with 1,393 curated defense-related genes (see the Materials and Methods section for details) and found that DCGs were significantly enriched in defense-related genes (hypergeometric test, *p*-value = 2.73 × 10^−09^). This observation highlighted the important functional roles of DCGs in plant defense responses.

For 129 plant defense-related DCGs, some have already been proved to play vital roles in plant immunity to *P. syringae*. For example, *FLS2*, which encodes an LRR receptor-like kinase involved in the perception of flagellin in *Arabidopsis*^37^, was differentially coexpressed with 229 genes. The observation reflected that the coexpression between *FLS2* and these 229 genes were extensively altered after *P. syringae* infection. Other important defense-related genes included the R gene *ADR1*, a JA synthetase *JAR1*, and so on. Notably, the DCG with the largest number of differential coexpression events was AT3G03440, which was involved in 1,001 differential coexpression gene pairs. AT3G03440 encodes a chloroplast-located armadillo (ARM) repeat family protein, but its physiological function remains elusive. Even so, some studies have suggested their potential functional importance in many biological processes, including disease resistance^38^,^39^. Through analyzing its 1,001 differentially coexpressed partners, “defense response” was found to be over-represented (hypergeometric test, *p*-value = 4.39 × 10^−3^, Table S3B). Based on these observations, AT3G03440 may be a good candidate to study plant immune responses to *P. syringae*.

### Differential coexpression analysis provides additional information that is complementary to differential expression analysis

Differential expression analysis is a routine method to conduct microarray data analysis. Generally, differential expression analysis treats each gene separately and does not consider its relationship with potential interaction partners. In this context, it is interesting to decipher the relationship between differential coexpression and differential expression based on the transcriptional dataset used in this work.

**Figure 2:**
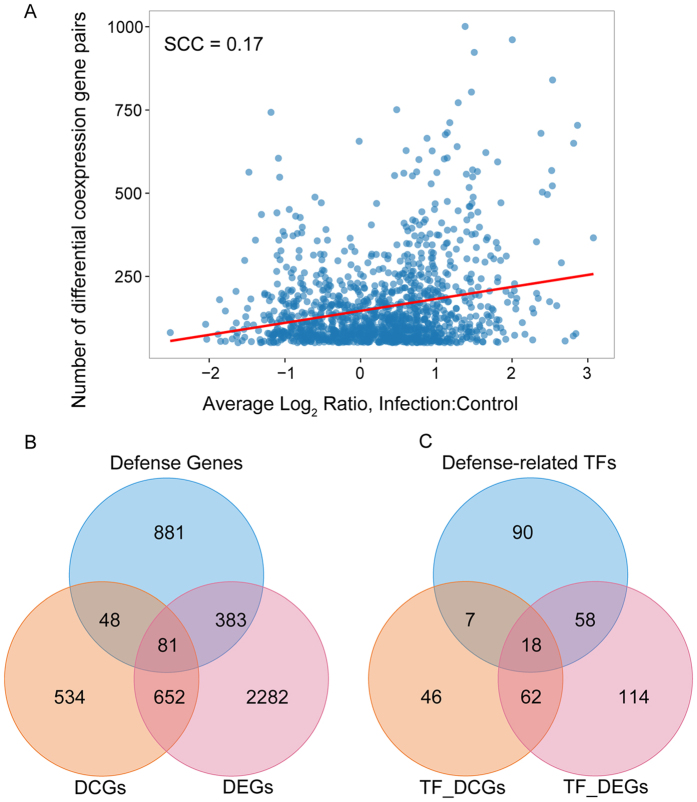
The relationship between differential expression and differential coexpression. **(A)** Differential expression and differential coexpression are weakly correlated. Differential expression is measured by the average log_2_-fold change across different time points. Each node indicates a DCG, and there are 1,315 DCGs in total. The red line shows linear fit, and the reported SCC shows the correlation between the two measures. **(B)** Overlap among DCGs, DEGs and plant defense-related genes. **(C)** Overlap among DCGs, DEGs and plant defense-related genes in the context of TFs. TF_DCGs represent differentially coexpressed TFs, whereas TF_DEG_S_ denote differentially expressed TFs.

We first calculated the magnitude of differential expression (measured by average log_2_-fold change) for each DCG (Table S2). Then, we compared the magnitude of differential expression with the number of differentially coexpressed gene pairs on the identified 1,315 DCGs. As shown in Fig. 2A, the correlation between these two measures was generally weak (SCC = 0.17). The result was consistent with the previous observation that differential expression analysis sheds little light on differential coexpression analysis^27^. We also compared 1,315 DCGs with 3,398 DEGs (Table S4) detected using the R package maSigPro^40^ (see the Materials and Methods section). The overlap between DCGs and DEGs was generally large, but there were 582 DCGs that were not detected as differentially expressed by maSigPro (Fig. 2B, Table S2). Moreover, we also calculated the overlap between DEGs and DCGs from another two datasets, *i.e.*, the dataset of *G. orontii* infection (GSE5686) and the dataset of *B. cinerea* infection (GSE29642). Using the same analysis procedures performed on GSE56094, we obtained 154 DEGs and 404 DCGs from GSE5686, and 4,817 DEGs and 1,676 DCGs from GSE29642. DCGs from GSE5686 and GSE29642 were both enriched for defense-related genes (hypergeometric test, *p*-value < 2.26 × 10^−16^ for DCGs from GSE5686 and *p*-value = 2.27 × 10^−14^ for DCGs from GSE29642). Despite the high overlap between DCGs and DEGs, we also observed that many DCGs could not be detected by differential expression analysis (Figure S2). These analyses further revealed the general importance of DCGs in plant defenses.

TFs are major players in regulating transcriptional reprogramming during plant immunity^41^. Therefore, we analyzed the relationship among DCGs, DEGs and plant defense-related genes in the context of TFs. In total, we obtained 1,996 TFs from the *Arabidopsis* Gene Regulatory Information Server (AGRIS)^42^ and the Plant Transcription Factor Database (PlantTFDB)^43^. As shown in Fig. 2C, DCGs and DEGs were both significantly enriched in defense-related TFs (hypergeometric test, *p*-value = 1.28 × 10^−4^ for TFs from DCGs and *p*-value < 2.20 × 10^−16^ for TFs from DEGs), indicating that TFs from DCGs or DEGs were important in plant immune responses. In total, 53 (40%) DCG-unique TFs were not defined as DEG, 7 of which participate in plant immune responses (Table S2).

The above comparative analyses quantified the relationship between DCGs and DEGs and highlighted their roles in plant immunity. Consistent with a previous study^27^, it is reasonable that some genes not only tend to have different coexpression partners under different conditions but are also differentially expressed. It is worth mentioning that the purpose of such comparative analysis is not to argue which measure is better. Rather, we conclude that differential coexpression analysis is complementary to differential expression analysis, which can provide additional candidates for the study of plant immunity.

### Potential TFs regulate the observed differential coexpression

Direct identification of TFs involved in plant immunity through differential expression analysis is insufficient, given that TFs also tend to be stably expressed and regulated at the protein level^26^,^44^. The disruption of TF binding sites can affect the expression correlation between its targets, which is recognized as a potential regulatory mechanism of differential coexpression^16^,^17^,^45^. To account for this phenomenon, we explored the underlying regulatory mechanisms of differential coexpression in plant immune responses to *P. syringae* by integrating the known regulatory network data available in public databases.

After removing redundant interactions, we collected 50,824 TF-target binary interactions between 529 TFs and 22,793 target genes from the *Arabidopsis* Transcriptional Regulatory Map (ATRM)^46^, AGRIS^42^ and AthaMap^47^ (Table S5). In total, 13,879 interactions between 333 TFs and 5,371 targets were retained for further analysis by filtering out stably expressed genes under each condition (variance <0.2 in both conditions). We investigated the relationship between TFs and their targets to identify TFs that potentially regulate differential coexpression. For this assessment, we implemented a metric for prioritizing TFs that were putative regulators of differential coexpression during plant immune responses. It was based on the hypothesis that a TF of greater importance in regulating differential coexpression should have a greater number of its targets forming differentially coexpressed gene pairs^23^. We applied a binomial test (see the Materials and Methods section) to determine whether the number of differentially coexpressed gene pairs between targets of a TF was higher than expected.

Among 333 TFs in the regulatory network we examined, 30 TFs were identified with a significantly greater number of differentially coexpressed gene pairs formed between targets of each TF (Table 1). In fact, two of them were not designed on the microarray platform we analyzed, and 22 of them cannot be identified by traditional differential expression analysis (Figure S3), further demonstrating the power of our method. Moreover, 10 of 30 TFs have been reported to play important roles in plant immune system (Marked with asterisk in Table 1). For example, *ANAC019*, *ANAC055*, and *ANAC072* participate in COR-triggered systemic susceptibility to *P. syringae* pv. *maculicola* ES4326, and triple-mutant plants exhibited enhanced resistance to *P. syringae* ES4326^48^. *MYB51* regulates the biosynthesis of phytoalexin, which is a critical defense metabolite in *Arabidopsis*^49^. In addition, *DREB1A*, *DREB1B* and *DREB1C* (also referred to as *CBF1*, *CBF2* and *CBF3*) from the DREB subfamily A-1 of ERF/AP2 TF family were all detected as TFs regulating differential coexpression. All of these three TFs positively regulate plant tolerance to cold stress^50^, but their roles in plant immunity have not been explored. Considering the potential crosstalk between plant responses to biotic and abiotic stresses^51^, these genes can serve as important candidates for further experimental validation.

For these 20 TFs with unknown roles in plant immunity, we assessed whether any indirect evidence is available indicating that they may be involved in plant immunity. By analyzing their targets, we found that the targets of 9 TFs are significantly enriched with defense-related GO terms (Table S6), suggesting a potential role for those TFs in regulating defense-related gene network. For example, the MADS TF *AGL15* was not differentially expressed between control and infection conditions (Figure S4A). However, the expression correlations between one of its targets (*HYS1*) and the other 87 targets were significantly increased after infection by *P. syringae* (Figure S4B). Interestingly, those targets regulated by *AGL15* are enriched in defense-related functions (Table S3C). The role of *HYS1* in plant immunity has been previously studied^52^, but how it is regulated is not clear. Based on the fact that the promoter of *HYS1* can bind with *AGL15*^42^ and the increased coexpression between HYS1 and other targets of AGL15, it is reasonable to assume *AGL15* may play a role when *Arabidopsis* is infected by *P. syringae*.

### Extensive gene coexpression rewiring in metabolic pathways during plant immune responses

**Figure 3:**
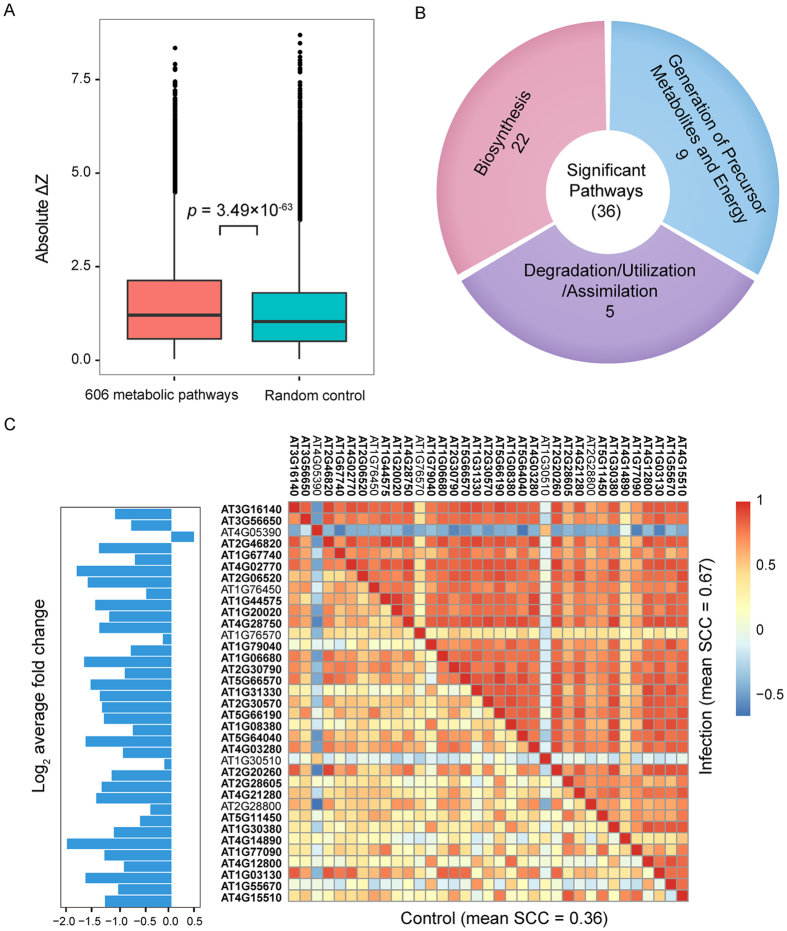
Differential coexpression in metabolic pathways. **(A)** The distribution of difference in correlation of 58,158 intra-pathway gene pairs (red) and 58,158 random gene pairs (green). **(B)** The categories of dysregulated pathways with significantly changed correlations between control and infection groups. **(C)** Photosynthesis light reactions pathway (PWY-101). The mean correlation of gene pairs in photosynthesis light reactions was 0.36 in the control group and increased to 0.67 after pathogen infection. Average log_2_-fold change values of the 35 metabolic genes are presented in the bar plot on the left. The 30 DEGs detected using the R package maSigPro are indicated in bold.

From the GO annotation of DCGs, we noticed that metabolic processes were significantly overrepresented (Table S3A). Plant metabolism constitutes integral part of the plant immune system. The primary metabolic pathways, such as photosynthesis, photorespiration and TCA cycle, provide the energy required for the defense responses^53^,^54^. The indispensable contribution of secondary metabolites to plant immunity, such as phytoalexin and various phenolic compounds, has also been well established^55^,^56^,^57^. Considering the important role of metabolites in plant immunity, we wonder how the coexpression pattern changes within metabolic pathways during plant immune responses to *P. syringae*. For this purpose, we first collected 606 metabolic pathways from the AraCyc database (V13.0) deposited in the Plant Metabolic Network (PMN) project^58^. Then, we analyzed the difference in correlation between infection and control for all possible gene pairs from a common pathway. We found that the absolute differences in correlation of metabolic gene pairs were larger for a pathway compared with a group of randomly selected genes (Fig. 3A, Wilcoxon test, *p*-value = 3.49 × 10^−63^). Based on this finding, we hypothesized that the coordination of metabolic genes in the same metabolic pathway was widely influenced during plant immune responses to *P. syringae*.

Then, we were interested in which metabolic pathway significantly changes coexpression patterns between its genes during plant immune responses to *P. syringae*. For this purpose, we assigned a pathway score to each pathway by measuring the difference in coexpression between control and infection conditions (see the Materials and Methods section). A larger pathway score indicated a larger difference in coexpression for the pathway. We used the concept of dysregulated pathways^59^ to represent those pathways. In total, 36 dysregulated pathways were identified (Table 2) and divided into three categories according their super pathways in PMN, including biosynthesis, degradation/utilization/assimilation, and generation of precursor metabolites and energy (Fig. 3B). As a comparison, the Gene Set Enrichment Analysis (GSEA) method^60^ was used to identify metabolic pathways with significantly changed expression levels, and 80 metabolic pathways were identified (nominal GSEA *p*-value < 0.05, Table S7). GSEA is a statistical method used to determine whether predefined gene sets (*i.e.*, metabolic pathway in our work) are differentially expressed between two conditions. Compared with GSEA, our method aims to detect dysregulated pathways with changed expression correlation rather than changed expression levels. For 36 dysregulated metabolic pathways detected using our method, only 16 pathways were revealed by GSEA. These results suggest that differential coexpression can provide new predictions for the identification of important pathways involved in plant immunity compared with the method only considering differential expression.

We further explored the relationship between dysregulated pathways and differentially expressed pathways. Regarding the 16 pathways detected as significant by both of our method and GSEA, it is possible that the coordinated changes in expression of genes in the pathway resulted in the altered gene coexpression. For example, the metabolic pathway “photosynthesis light reactions” (Pathway ID: PWY-101) was identified as differentially expressed as well as dysregulated (*i.e.*, differentially coexpressed). The average correlation among genes within this pathway increased from 0.36 to 0.67 after pathogen infection, whereas most genes in this pathway (30 out of 35) were significantly down-regulated (Fig. 3C). Increasing evidences showed that photosynthesis is inhibited during immune responses^61^,^62^. Not surprisingly, the original study reported the down-regulation of genes located in chloroplasts during plant responses to *P. syringae*^30^. Therefore, genes in PWY-101 were coordinately down-regulated during plant immune responses (Figure S5).

For the 20 pathways specifically identified by our method (not differentially expressed between two conditions), we also observed some plant defense-related pathways. For example, the glutathione biosynthesis pathway (Pathway ID: GLUTATHIONESYN-PWY) was detected as a dysregulated pathway by our method. There were two enzymes in GLUTATHIONESYN-PWY, and the coexpression between them was increased from −0.41 (control) to 0.85 (infection) (Figure S6). Glutathione participates in detoxification and signaling in plant defense against biotic and abiotic stresses^63^. The abundance of glutathione increases in the plants infected with avirulent pathogens or insect *Mayetiola destructor*^64^. The increased correlation of GLUTATHIONESYN-PWY under pathogen infection may favor the production of glutathione, which contributes to plant immune responses to *P. syringae*.

Pathways with roles in plant immunity that have not been reported may serve as good candidates for further analysis. Our expression correlation-based method found that aerobic respiration III (Pathway ID: PWY-4302) was dysregulated during plant immune responses to *P. syringae*, but the GSEA analysis recognized it as non-differentially expressed. Within this pathway, a majority of genes (93%) displayed a stable expression pattern in control and infection conditions. Regarding the expression correlation between genes in this pathway, we observed large changes in correlation between a subset of genes in this pathway (Figure S7). Although genes in the subset exhibited minimal changes in gene expression, the expression correlation between these genes significantly changed. In general, the generation of energy and release of ROS during aerobic respiration may indicate the potential role of this pathway in plant immunity^65^. Further validation is needed to decipher how these coexpression changes influence plant immune responses to *P. syringae*.

### Comparison of current work with previous studies

Our current work is primarily based on the dataset of GSE56094, and the resultant new findings have been clearly described in the aforementioned sections. Here, we focus on discussing the technical differences between our work and the original studies based on the same microarray data^30^,^31^,^32^. In these original studies, de Torres Zabala *et al*. focused on the expression of nuclear-encoded chloroplast-targeted genes^30^. The group further analyzed the expression pattern of genes related to jasmonate signaling^31^. Lewis *et al*. aimed to determine the difference between transcriptional reprogramming associated with microbial-associated molecular pattern-triggered immunity and effector-triggered susceptibility^32^. In contrast to these original studies, in this work GSE56094 was used to conduct differential coexpression analysis. A series of computational analyses were performed in our work, including DCG analysis, metabolic pathway analysis and transcriptional regulation analysis.

As reported in Lewis *et al*., three techniques were utilized to identify DEGs^32^. We also compared the list of 9,782 DEGs between mock-infiltrated leaves and DC3000-infiltrated leaves identified by the original study of Lewis *et al*.^32^ with 1,315 DCGs detected in this work. We found that 238 out of 1,315 DCGs were not included in the DEG list of Lewis *et al*. (2015) (Table S2), further indicating the complementarity between DEGs and DCGs. Regarding the module/pathway analysis, Lewis *et al*. used coexpression-based clustering method to identify gene clusters coexpressed within each treatment (mock treatment, infection by virulent *P. syringae* or avirulent *P. syringae* with *hrpA* mutant)^32^. In our analysis, we first demonstrated extensive gene coexpression rewiring in metabolic pathways during plant immune responses and then identified 36 dysregulated metabolic pathways with significant changes in expression correlation between normal growth and pathogen infection (Table 2). This finding differs from the coexpression-based clustering analysis performed by Lewis *et al*. With respect to the coregulation analysis, Lewis *et al*. aimed to predict the specific regulation of pathogen-response genes, whereas we sought to identify potential TFs regulating the observed differential expression during plant immune responses. In summary, our current analyses exhibit technical complementarity to the three original studies based on the same dataset GSE56094 and thus provide new insights into our understanding of plant immunity.

## Conclusions

Plants are challenged by various pathogens during all phases of their development. To effectively defend against these infections, plants have evolved a powerful immune system, and extensive transcriptional reprogramming exists during this process. In this work, we initiated a study to investigate *Arabidopsis* immune responses to the bacterium *P. syringae* through differential coexpression analysis. We concluded that differential coexpression was a common phenomenon during plant immunity. Moreover, we found that DCGs, which are complementary to DEGs, also played important roles in plant immune responses. By integrating regulatory networks into our analysis, we identified several TFs that may regulate this differential coexpression during plant immune responses. We noticed extensive differential coexpression of metabolic genes during plant immunity and identified 36 dysregulated metabolic pathways. In the future, the transcriptional data measuring plant responses to pathogen infection will be increasingly available, allowing us to perform the current analysis using sizable expression data and achieve more robust results. In the meantime, the flourish of transcriptional data will also allow us to answer some specific biological questions in the context of differential coexpression. For instance, comparative analysis of differential coexpression during plant immune responses to different pathogens should be an important topic. It is our expectation that the differential coexpression analysis can boost the study of plant immune response-related transcriptomics and provide new insights into deciphering the molecular mechanisms of plant-pathogen interactions.

## Materials and Methods

### Data collection

Normalized expression dataset (GEO accession number: GSE56094) were directly downloaded from the GEO database^33^. GSE56094 was designed to measure *Arabidopsis* gene expression at a high resolution under three conditions: mock treatment, or infections with either *P. syringae* pv. *tomato* DC3000 or the corresponding nonpathogenic *hrpA* mutant^30^,^31^,^32^. The detailed experimental procedures of the pathogen treatments and microarray data processing were documented by Lewis *et al*.^32^. Probe sets were mapped to their corresponding AGI (Arabidopsis Genome Initiative) gene identifiers according to the annotation file from GEO, and replicated probes of the same gene were averaged. After data processing, we obtained 23,974 unique genes. *Arabidopsis* metabolic pathways were obtained from the AraCyc database^58^ at PMN (http://www.plantcyc.org/). Genes in each pathway were filtered using expression data, and only pathways with genes detected on the microarray were retained for analysis. *Arabidopsis* transcriptional regulatory networks were collected from ATRM^46^, AGRIS^42^ and AthaMap^47^, and TFs were collected from PlantTFDB^43^ and AGRIS. For predicted regulatory networks from AthaMap, only TFs with binding sites determined by pattern-based screenings were considered in our work.

Plant defense-related genes were collected through analyzing annotation information from the TAIR GO.Slim files and the literature^41^. We first downloaded the GO.Slim files from the FTP site of TAIR (ftp://ftp.arabidopsis.org/home/tair/Ontologies/). Then, for each record in the annotation file, if the description of a gene contained at least one of the following biological process keywords, *i.e.*, “systemic acquired resistance”, “disease resistance”, and “immune” and “defense”, we selected the gene as a plant defense-related gene. Moreover, Tsuda and Somssich recently provided a list of TFs associated with plant immunity in their review article^41^. These TFs were also assigned as defense-related genes. Furthermore, collected defense-related genes were filtered using expression data, and only genes detected on the microarray were reserved.

### Detection of differentially coexpressed gene pairs

Differentially coexpressed gene pairs were detected using the R package DiffCorr^22^, and the workflow is summarized as follows (Fig. 1A). First, the obtained microarray data were divided into infection and control groups. Prior to the calculation of the coexpression correlation coefficient, we removed genes with expression variance less than 0.2 across both infection samples and control samples. Then, for each group, SCCs and Benjamini-Hochberg^66^ adjusted *p*-values for all possible gene pairs were calculated. Here, a gene pair was defined as significantly coexpressed if its corrected *p*-value was less than a given cutoff of 10^−8^. Next, the difference in correlation of a gene pair (*i, j*) between infection and control conditions was evaluated by the following equation:


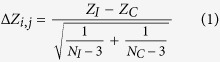


where *N*_*I*_ and *N*_*C*_ was the sample number in the infection and control groups, respectively; *Z*_*I*_ and *Z*_*C*_ were Fisher’s transformation of SCC between the gene pair (*i, j*) under infection and control conditions, respectively, and had been used in previous differential coexpression analysis^22^,^27^. The Δ*Z* followed a Gaussian distribution^22^, and the associated *p*-value was evaluated based on the distribution and corrected using the Benjamini-Hochberg correction. If the corrected *p*-value is less than 10^−8^, the difference in correlation was regarded as significant. Finally, we defined a pair of genes as differentially coexpressed if they exhibited both a significant difference in correlation and a significant correlation under at least one of the two conditions.

### Identification of DCGs

In our work, DCGs were defined as genes whose expression correlations with many other genes were significantly changed between infection and control conditions. The binomial probability model was used to identify DCGs^67^. Given that the probability of observing a “differential coexpression gene pair” was *p* (*p* = 5.41 × 10^−3^ in this work), the *p*-value of differential coexpression for the gene *i* with *k*_*i*_ differentially coexpressed gene pairs was evaluated as follows:


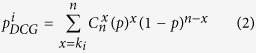


where *n* was the number of genes used to identify differentially coexpressed gene pairs. Then, 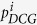 was corrected using the Benjamini-Hochberg correction for multiple testing.

### Identification of DEGs

The Bioconductor package maSigPro was used to identify DEGs^40^. The obtained 104 samples of 52 infection samples and 52 control samples were used as input. The maSigPro method follows a two-step regression strategy to identify DEGs from time-course microarray data. Regarding the parameters of maSigPro, Q value and “alfa” were set as 0.01, and a default R^2^ threshold of 0.6 was adopted. After MaSigPro analysis, a cutoff of abs (log_2_-fold change) ≥0.58 (*i.e.*, at least 1.5-fold change) was applied to designate genes as differentially expressed.

### GO enrichment analysis

GO enrichment analysis was performed using BiNGO 3.02 in Cytoscape with the “GO Biological Process” category^68^. Using the whole annotation as the reference set, over-represented terms were selected with a significance level of 0.05 (hypergeometric test) after Benjamini-Hochberg correction.

### Statistical test for TFs regulating differential coexpression

Our analyses were based on the assumption that if the targets of a TF tend to form differentially coexpressed gene pairs, the TF must be more important in regulating differential coexpression^23^. Given that the probability of observing a “differential coexpression gene pair” was *p* (*p* = 5.41 × 10^−3^ in this work), the probability of forming at least *e* differential coexpression gene pairs between TF targets by chance was:


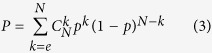


If a TF had *n* targets, *N* was equal to *n* × (*n* − 1)/2. A correction for multiple testing using Benjamini-Hochberg method was then applied to the vector of *P*-values for all TFs.

### Calculation of pathway score for metabolic pathways

To identify dysregulated pathway, we first calculated average difference in expression correlation for all possible gene pairs within the given pathway. The pathways associated with only one gene were discarded from this analysis. For a pathway *i* with *m* genes, there were a total *m* × (*m* − 1)/2 possible gene pairs in this pathway, and we defined this gene pair set as *G*_*i*_. The pathway score was calculated as follows:


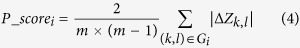


where Δ*Z*_*k,l*_ was calculated using Eq. 1 representing difference in correlation for the gene pair (*k, l*) from *G*_*i*_.

Then, we generated a random pathway score distribution for each pathway to evaluate the significance of the pathway score. For a pathway with *m* genes, we randomly selected *m* genes from the microarray and calculated a pathway score using Eq. 4. We repeated the process 1,000 times to form a random pathway score distribution. An empirical *p*-value for the pathway was calculated as the fraction of permutation values that were greater than or equal to the observed value and corrected for multiple testing using the Benjamini-Hochberg correction. Finally, a pathway was defined as a dysregulated pathway in plant immune responses to *P. syringae* if its corrected *p*-value was less than a given cutoff.

GSEA was also used to identify metabolic pathways with significantly changed expression levels and was performed using the GSEA software^60^. The minimum and maximum gene set sizes were set to 5 and 1,000, respectively. The metric for ranking genes was set to “Diff_of_Classes”. 1000 permutations were used to calculate *p*-value and the permutation type was set to “Gene_set”. All other parameters were set as default. A cutoff of 0.05 was used to define statistical significance.

## Additional Information

**How to cite this article**: Jiang, Z. *et al*. Differential Coexpression Analysis Reveals Extensive Rewiring of *Arabidopsis* Gene Coexpression in Response to *Pseudomonas syringae* Infection. *Sci. Rep.* **6**, 35064; doi: 10.1038/srep35064 (2016).

## Supplementary Material

Supplementary Information

Supplementary Table S1

Supplementary Table S2

Supplementary Table S3

Supplementary Table S4

Supplementary Table S5

Supplementary Table S6

Supplementary Table S7

## Figures and Tables

**Table 1:** Transcription factors with targets that significantly form differential coexpression gene pairs.

Gene^a^	Symbol	Target number	Differential coexpression gene pairs	*p*-value^b^
AT5G13790	*AGL15*	1,520	9,150	<2.20 × 10^−16^
AT5G61850	*LFY3*	1,499	7,353	<2.20 × 10^−16^
AT4G16110*	*ARR2*	370	585	<2.20 × 10^−16^
AT2G43010	*PIF4*	726	1,995	<2.20 × 10^−16^
AT1G24260	*SEP3*	1,689	11,245	<2.20 × 10^−16^
AT5G07100	*WRKY26*	150	133	1.51 × 10^−13^
AT3G16857	*ARR1*	222	238	2.10 × 10^−13^
AT2G46130	*WRKY43*	132	109	6.62 × 10^−13^
AT5G22570*	*WRKY38*	133	109	1.49 × 10^−12^
AT5G11260	*HY5*	372	511	6.38 × 10^−08^
AT4G27410*	*ANAC072*	8	5	7.98 × 10^−08^
AT4G25490	*DREB1B*	16	8	2.39 × 10^−07^
AT4G25480	*DREB1A*	40	19	2.89 × 10^−07^
AT4G36990*	*TBF1*	1,090	3,705	4.00 × 10^−07^
AT3G14230*	*RAP2.2*	278	294	4.40 × 10^−06^
AT1G13450	*GT-1*	115	68	1.12 × 10^−05^
AT2G20180	*PIF1*	356	434	4.02 × 10^−04^
AT4G27330	*SPL*	3	1	4.02 × 10^−04^
AT1G27730*	*ZAT10*	5	1	5.57 × 10^−03^
AT4G25470	*DREB1C*	18	4	7.37 × 10^−03^
**AT5G05410**	*DREB2A*	32	8	9.05 × 10^−03^
AT1G18570*	*MYB51*	6	1	1.05 × 10^−02^
AT3G15500*	*ANAC055*	6	1	1.05 × 10^−02^
AT5G11590	*TINY2*	20	4	1.55 × 10^−02^
AT1G52890*	*NAC019*	7	1	1.89 × 10^−02^
AT1G69490*	*ANAC029*	25	5	2.32 × 10^−02^
AT2G16770	*bZIP23*	13	2	2.84 × 10^−02^
AT5G56110	*MYB80*	129	62	3.95 × 10^−02^
AT4G35040	*bZIP19*	14	2	3.95 × 10^−02^
AT3G24050	*GATA1*	307	304	3.95 × 10^−02^

^a^DEGs are in bold. TFs that are vital for *Arabidopsis* defense are marked with an asterisk.

^b^*p*-values are corrected using the Benjamini-Hochberg correction.

**Table 2:** Dysregulated pathways with significantly changed expression correlations.

Pathway ID^a^	Pathway Name	#Gene	#DEG^b^	P_score^c^	*P*-value^d^
SUCSYN-PWY^*^	Sucrose biosynthesis I	45	20	1.79	<1.00 × 10^−03^
PWY-5046	2-Oxoisovalerate decarboxylation to isobutanoyl-CoA	10	2	2.34	<1.00 × 10^−03^
CITRULBIO-PWY	Citrulline biosynthesis	26	7	2.02	<1.00 × 10^−03^
PWY-101^*^	Photosynthesis light reactions	35	30	2.60	<1.00 × 10^−03^
THRESYN-PWY	Threonine biosynthesis	19	4	2.00	<1.00 × 10^−03^
PWY66-21	Ethanol degradation II	22	9	1.95	<1.00 × 10^−03^
GLYCLEAV-PWY^*^	Glycine cleavage	10	5	2.36	<1.00 × 10^−03^
PWY-7328	Super pathway of UDP-glucose-derived O-antigen building blocks biosynthesis	28	5	1.99	<1.00 × 10^−03^
PWY-5464^*^	Super pathway of cytosolic glycolysis, pyruvate dehydrogenase and TCA cycle	110	31	1.56	<1.00 × 10^−03^
PWY-622^*^	Starch biosynthesis	27	12	1.90	<1.00 × 10^−03^
CALVIN-PWY^*^	Calvin-Benson-Bassham cycle	37	25	1.87	<1.00 × 10^−03^
COLANSYN-PWY	Colanic acid building blocks biosynthesis	38	7	1.93	<1.00 × 10^−03^
PHOTOALL-PWY^*^	Oxygenic photosynthesis	72	55	2.14	<1.00 × 10^−03^
PWY-5004	Super pathway of citrulline metabolism	31	7	1.98	<1.00 × 10^−03^
PWYQT-4470	γ-Glutamyl cycle	3	0	4.84	<1.00 × 10^−03^
NONOXIPENT-PWY	Pentose phosphate pathway	12	6	2.43	<1.00 × 10^−03^
GLUTATHIONESYN-PWY	Glutathione biosynthesis	2	0	8.30	<1.00 × 10^−03^
PWY-5723^*^	Rubisco shunt	35	17	1.80	<1.00 × 10^−03^
CAROTENOID-PWY^*^	Super pathway of carotenoid biosynthesis	20	11	2.03	2.04 × 10^−02^
PWY-5805	Nonaprenyl diphosphate biosynthesis I	2	2	6.63	2.04 × 10^−02^
PWY-4302	Aerobic respiration III	56	4	1.63	2.04 × 10^−02^
PWY-6415	L-ascorbate biosynthesis V	23	5	1.99	2.04 × 10^−02^
PWY-2501	Fatty acid α-oxidation I	10	6	2.45	2.04 × 10^−02^
HOMOSERSYN-PWY	Homoserine biosynthesis	7	1	2.72	2.04 × 10^−02^
TYRFUMCAT-PWY^*^	Tyrosine degradation I	8	2	2.66	2.04 × 10^−02^
HEXPPSYN-PWY	Hexaprenyl diphosphate biosynthesis	2	2	6.63	2.04 × 10^−02^
COMPLETE-ARO-ARA-PWY^*^	Super pathway of phenylalanine, tyrosine and tryptophan biosynthesis	26	10	1.94	2.04 × 10^−02^
PWY-5114^*^	UDP-sugars interconversion	29	5	1.78	3.24 × 10^−02^
PWY-1422	Vitamin E biosynthesis	6	3	2.72	3.24 × 10^−02^
PWY-6444	Benzoate biosynthesis II	12	7	2.10	3.24 × 10^−02^
PWY-601*	Glucosinolate biosynthesis from tryptophan	25	7	1.78	3.24 × 10^−02^
PWYQT-4450^*^	Aliphatic glucosinolate biosynthesis, side chain elongation cycle	7	4	2.53	3.24 × 10^−02^
PWY-5783	Octaprenyl diphosphate biosynthesis	2	2	6.63	3.24 × 10^−02^
GLUTAMINDEG-PWY	Glutamine degradation I	18	5	1.99	3.24 × 10^−02^
TRPSYN-PWY^*^	Tryptophan biosynthesis	14	7	2.06	4.59 × 10^−02^
PWY-181*	Photorespiration	26	12	1.72	4.59 × 10^−02^

^a^The pathways detected as significant through the GSEA analysis are marked with asterisk.

^b^DEGs were detected using the R package maSigPro.

^c^P_scores are calculated using Eq. 4.

^d^*p*-values were corrected using the Benjamini-Hochberg correction.
