# Supplementary material for: Differential Coexpression Analysis Reveals Extensive Rewiring of Arabidopsis Gene Coexpression in Response to Pseudomonas syringae Infection
Source: Sci Rep. 2016 Oct 10;6:35064. doi: 10.1038/srep35064 (PMC5056366; doi:10.1038/srep35064)
Supplement: Supplementary Information [file srep35064-s1.doc]

**Supplemental Information**

Differential Coexpression Analysis Reveals Extensive Rewiring of Arabidopsis Genes Coexpression in Response to *Pseudomonas syringae* Infection

Zhenhong Jiang, Xiaobao Dong, Zhi-Gang Li, Fei He^*^ and Ziding Zhang*

^*^Corresponding authors (Z.Z.: Email: [zidingzhang@cau.edu.cn](mailto:zidingzhang@cau.edu.cn); F.H: plane83@gmail.com)


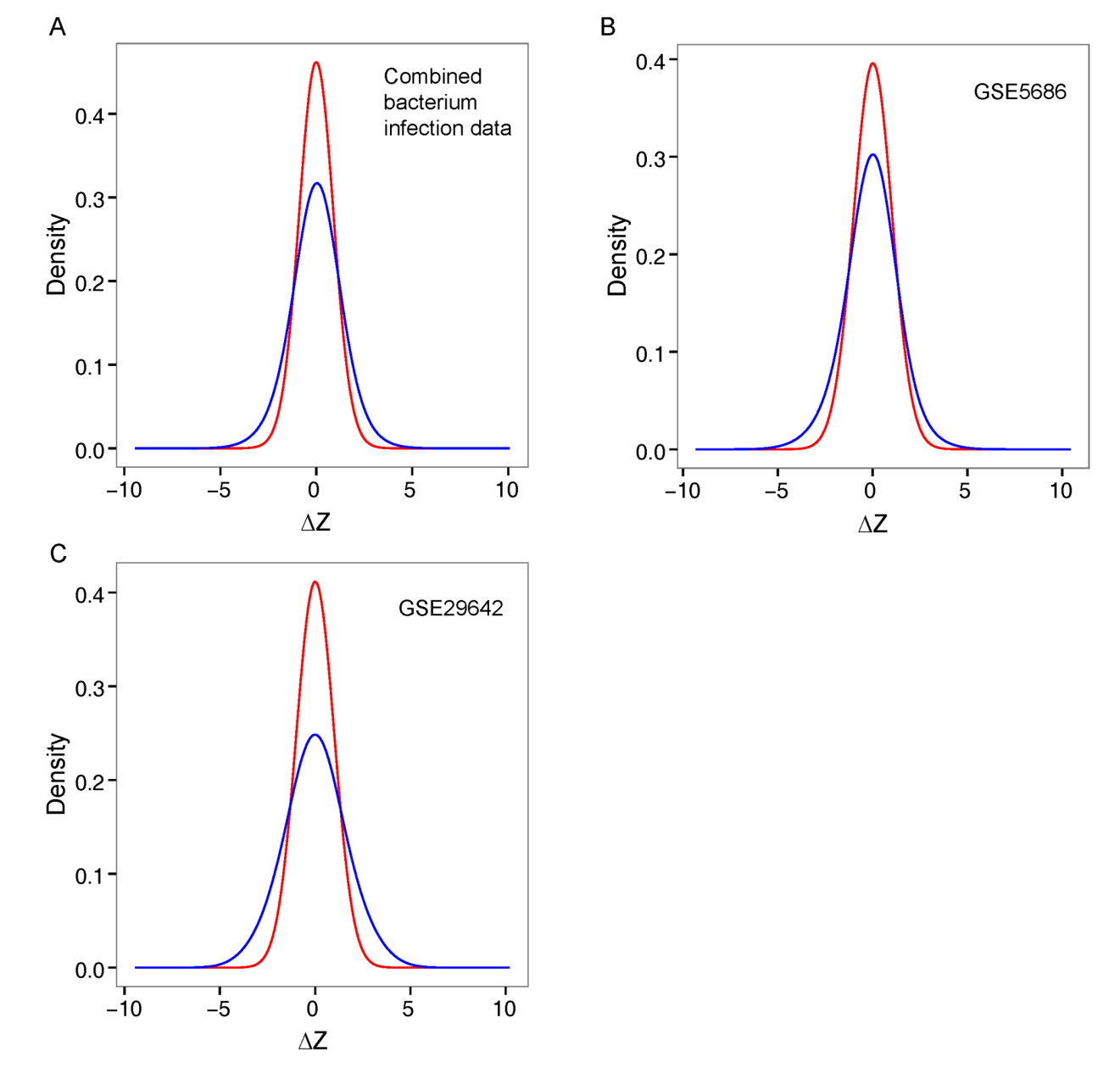


**Figure S1. The distribution of transformed difference in correlation for three datasets, i.e. *P. syringae* infection data, *G. orontii* infection data and *B. cinerea* infection data.** Blue lines denote real data, while red lines stands for shuffled data.(A) Combined bacterium infection dataset was composed of 50 samples with 25 infected samples and 25 control samples from 5 series, which measured gene expression after the infection of *P. syringae*. (B) GSE5686 consisted of 24 infected samples and 24 control samples, which measured gene expression after *G. orontii* infection. (C) GSE29642 was also composed of 24 infected and 24 control samples, which measured gene expression after *B. cinerea* infection. Compared to permutated data, all the real data from the three datasets have larger difference in correlation.


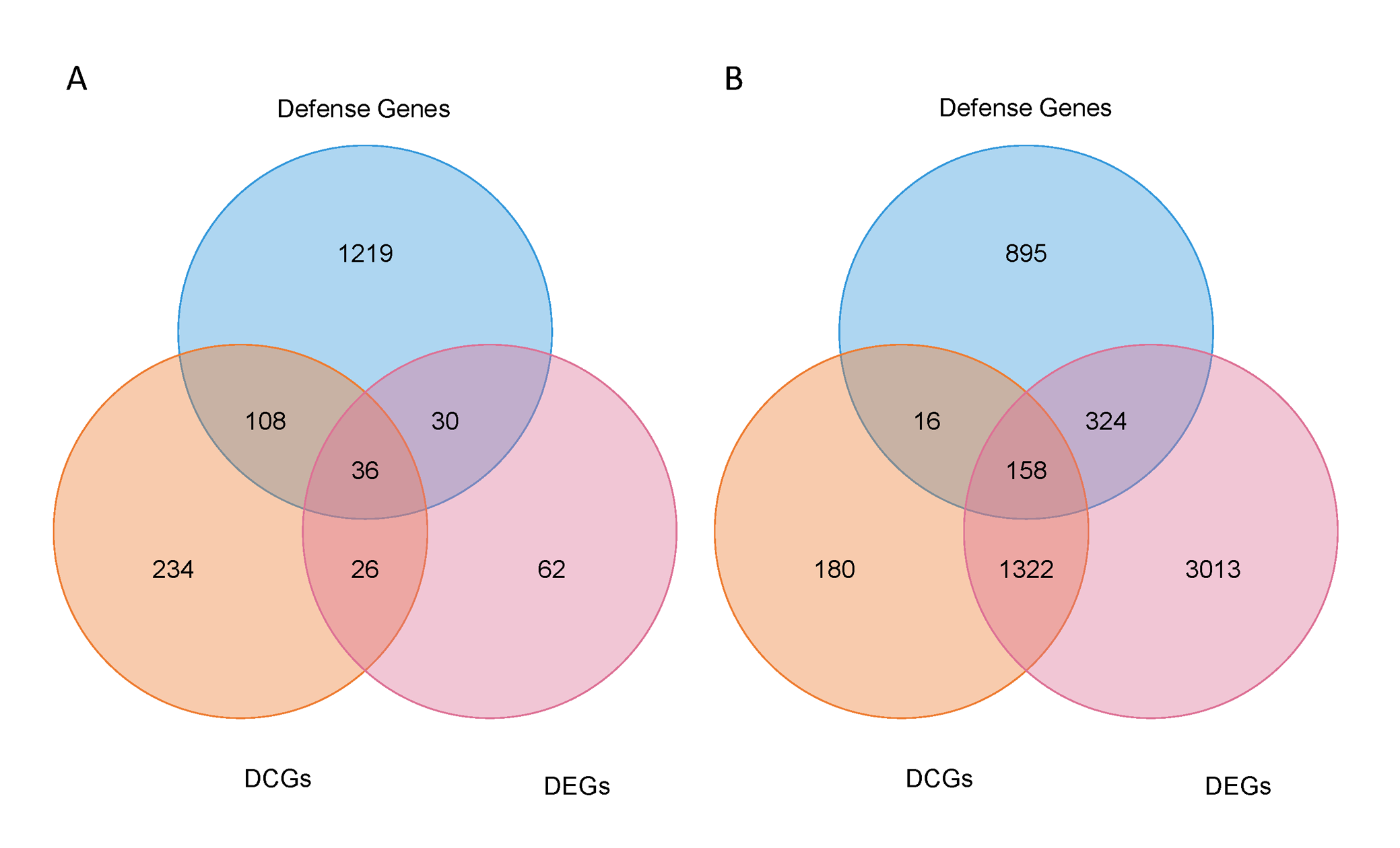


**Figure S2. Overlap between DCGs, DEGs and plant defense-related genes.** (A), DCGs and DEGs detected from *G. orontii* infection dataset GSE5686. (B), DCGs and DEGs detected from *B. cinerea* infection dataset GSE29642.


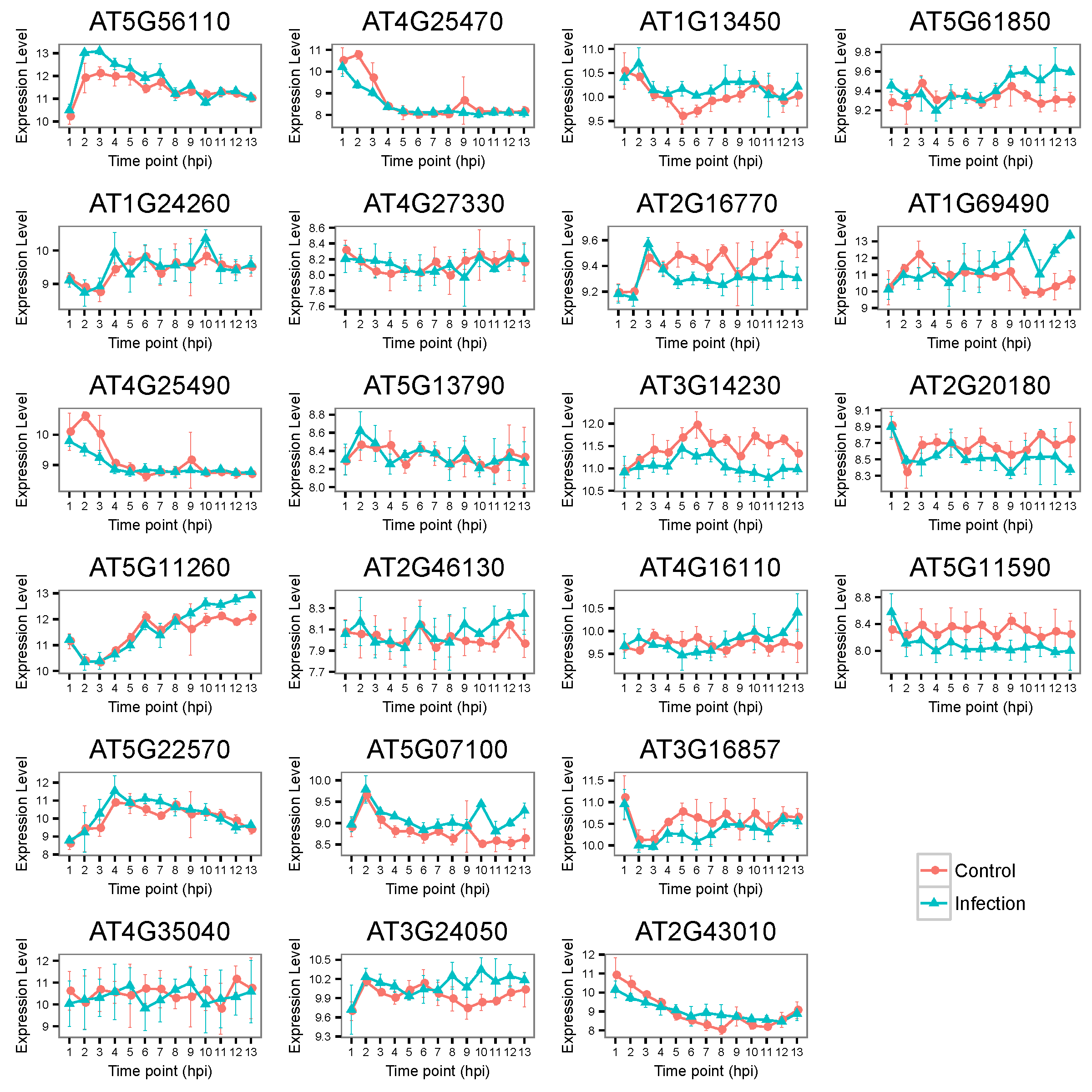


**Figure S3. The expression profiles of 22 TFs which cannot be identified using traditional differential expression analysis.** For each time point, replicated samples are averaged to obtain the final expression level. The error bars indicate standard deviations (SDs) among four biological replicates.


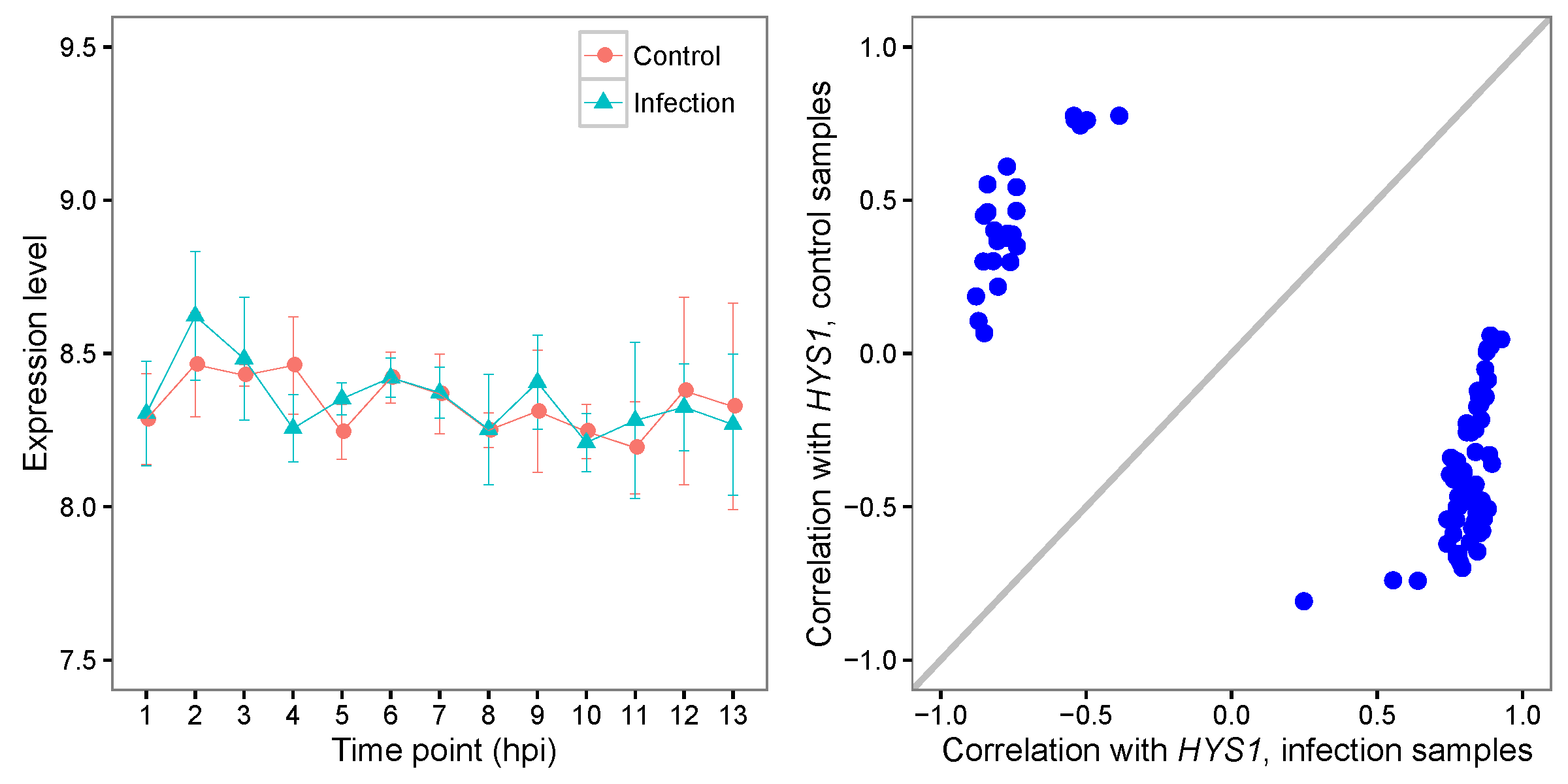


**Figure S4**. **The expression profile of** ***AGL15* and expression correlations between *HYS1* and the other 87 *AGL15* targets.** (A) For each time point, replicated samples are averaged to obtain the final expression level. The error bars indicate SDs among four biological replicates. *AGL15* is stably expressed in both control and infection conditions. (B) The expression correlations between *HYS1* and the other 87 *AGL15* targets are significantly changed between control and infection samples. Correlation is measured using the spearman correlation coefficient.


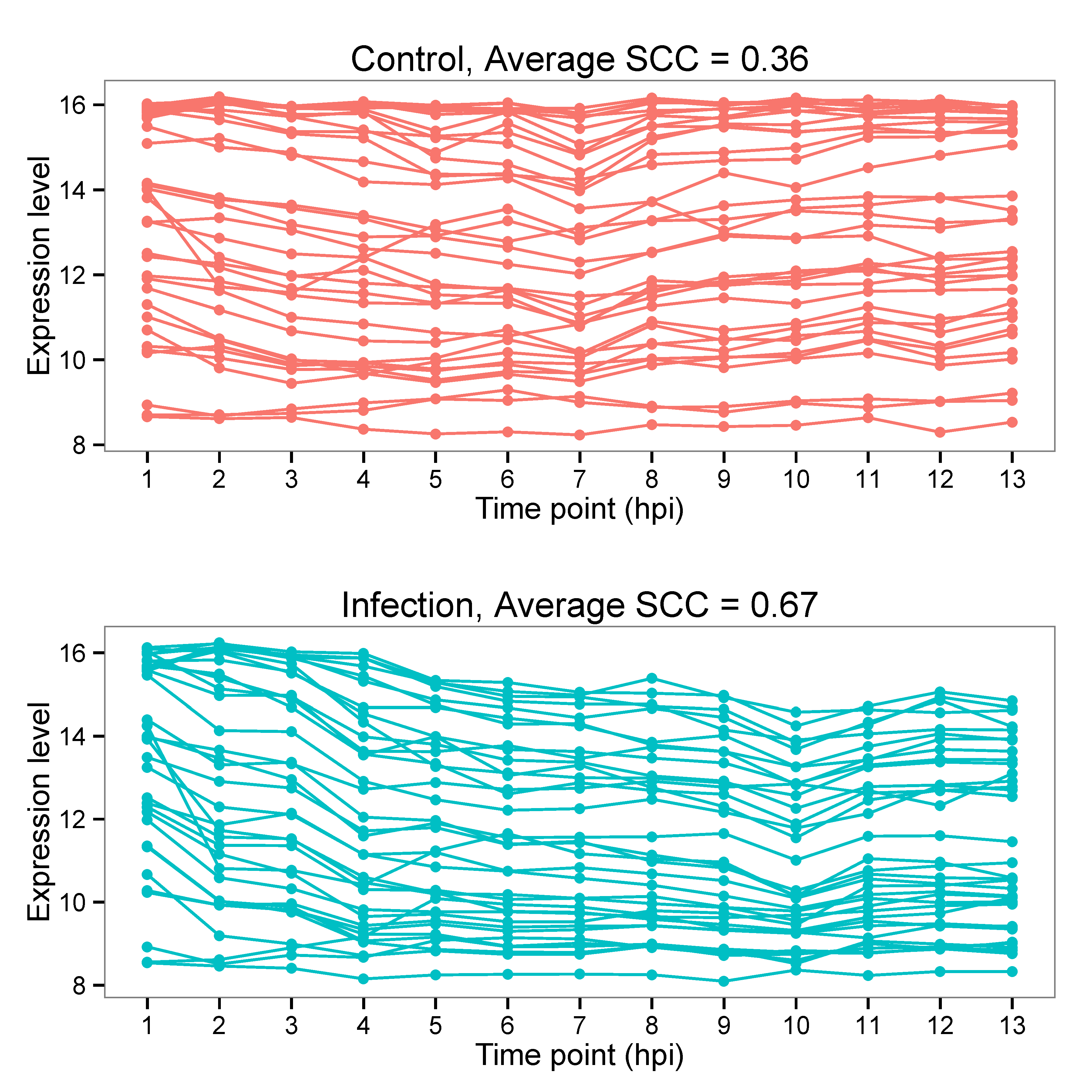


**Figure S5. The expression profiles of 35 genes in the PWY-101 pathway.** For each time point, replicated samples are averaged to obtain the final expression level. The average expression correlation (measured by SCC) between 35 genes in the PWY-101 pathway is increased from 0.36 (control) to 0.67 (infection).


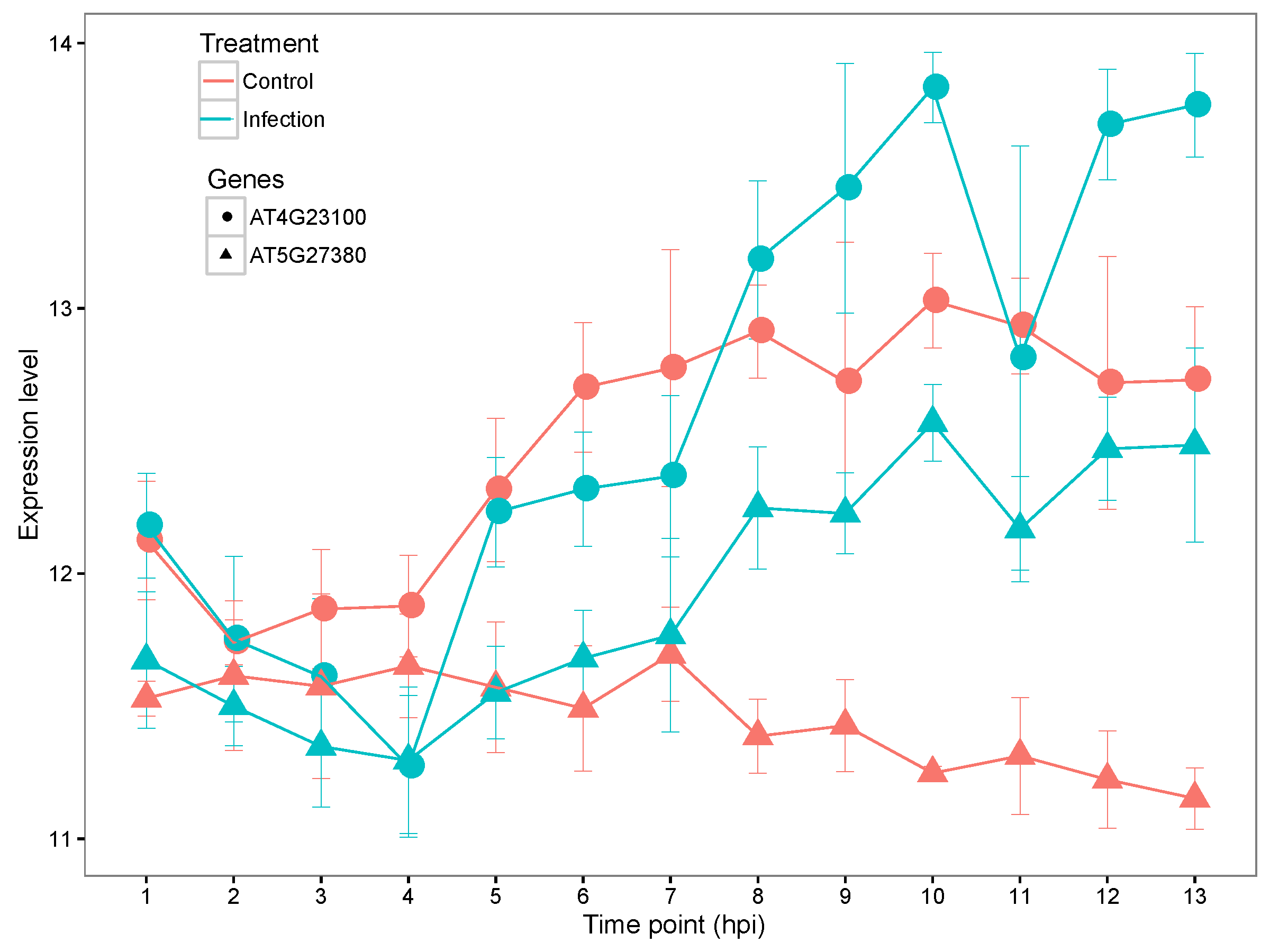


**Figure S6. The expression profiles of two genes in** **the GLUTATHIONESYN-PWY pathway.** For each time point, replicated samples are averaged to obtain the final expression level. The error bars indicate SDs among four biological replicates. Two genes (AT4G23100 and AT5G27380) are stably expressed in infection and control conditions. However, the coexpression between AT4G23100 and AT5G27380 is increased from -0.41 (control) to 0.85 (infection).


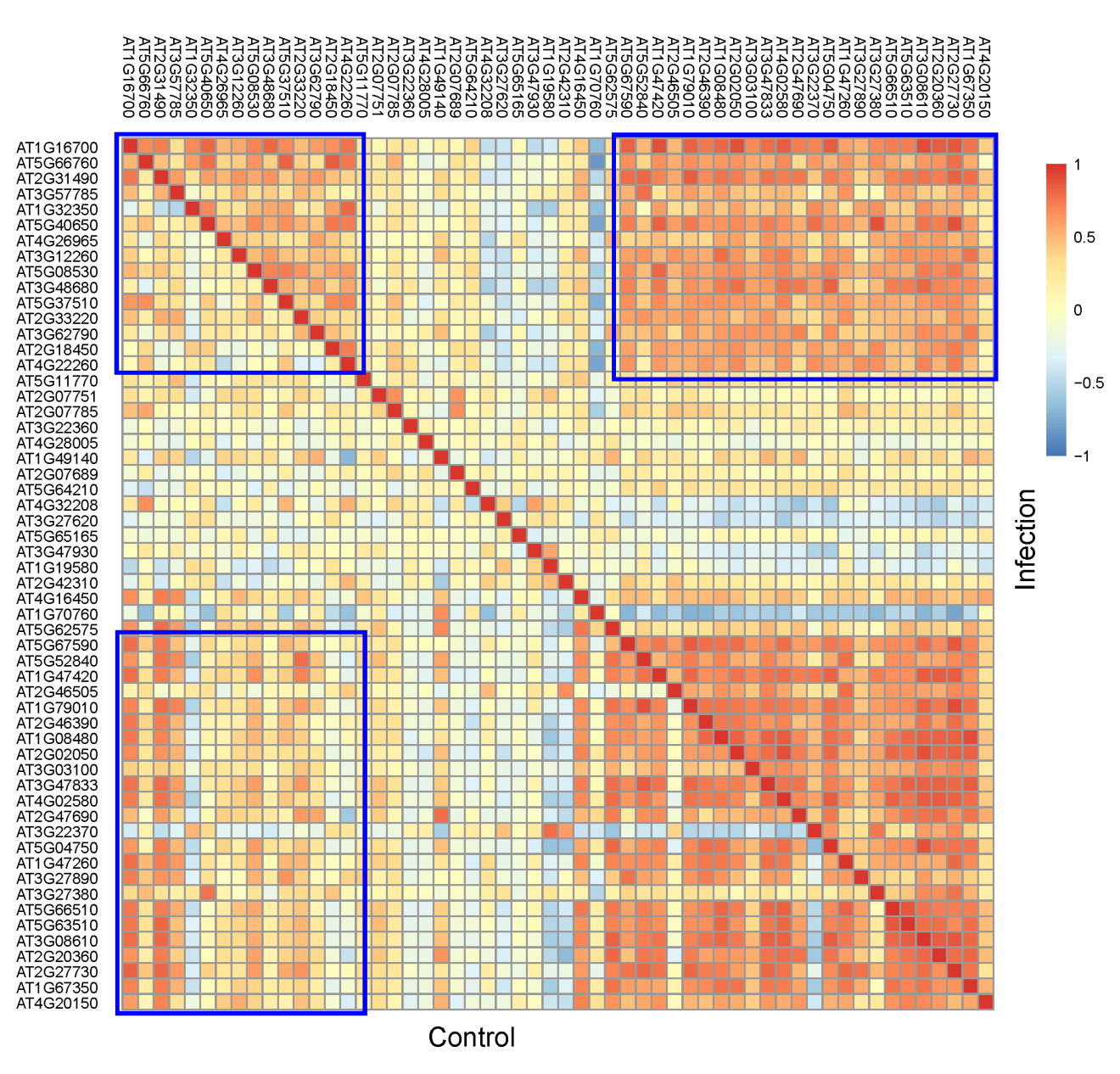


**Figure S7. Coexpression correlation between genes within the metabolic pathway, ‘aerobic respiration III’ (Pathway ID: PWY-4302).** The PWY-4302 is identified as dysregulated during plant immune response to *P. syringae*. The color scale represents the degree of correlation (measured using SCC) between two genes in PWY-4302. The upper triangular plot represents the SCC calculated using 52 infection samples and the lower triangular plot represents the SCC calculated using 52 control samples. Gene pairs in the regions marked with blue square show large changes in SCC between control and infection conditions.

**Table S1.** Three datasets measuring Arabidopsis gene expression responding to the infections by *P. syringae*, *G. orontii* and *B. cinerea*.

**Table S2.** The identified DCGs.

**Table S3.** Annotation results for DCGs, 1,001 genes which are differentially coexpressed with AT3G03440 and 9,150 differential coexpression gene pairs regulated by *AGL15*.

**Table S4.** DEGs detected using the R package maSigPro.

**Table S5.** Statistics of the TF-target interaction data collected from three different databases.

**Table S6.** Enriched GO terms for 20 TFs with unknown functions in plant immunity.

**Table S7.** Significant pathways detected using GSEA.

*Note that all Supplemental Tables are available in separated Excel files named Table_S1.xls, Table_S2.xls, Table_S3.xls, Table_S4.xls, Table_S5.xls, Table_S6.xls* and *Table_S7.xls.*
